# A Reliability Generalization Meta-Analysis of the Eating Attitudes Test 26 (EAT-26) Scale

**DOI:** 10.7759/cureus.73647

**Published:** 2024-11-13

**Authors:** Shaikha Janahi, Nayla Alkhater, Aysha Bucheer, Yasmeen Hashem, Khawla K Alothman, Alia Alsada, Maryam Bucheer, Haneen Jandeel, Dana AlJamea, Raghad Al Aqaili, Hadeel Ghazzawi, Haitham Jahrami

**Affiliations:** 1 Paediatric Surgery, Bahrain Defense Force Hospital, Kingdom of Bahrain, East Riffa, BHR; 2 Ophthalmology, King Hamad University Hospital (KHUH), Muharraq, BHR; 3 Paediatrics, King Hamad University Hospital (KHUH), Muharraq, BHR; 4 Family Medicine, Farwanyia Hospital, Farwanyia, KWT; 5 Psychiatry, King Hamad University Hospital (KHUH), Muharraq, BHR; 6 Psychiatry, Arabian Gulf University (AGU), Manama, BHR; 7 Psychiatry, Salmaniya Medical Complex, Manama, BHR; 8 Family and Community Medicine, School of Medicine, The University of Jordan, Amman, JOR; 9 Nutrition and Food Technology, School of Agriculture, The University of Jordan, Amman, JOR

**Keywords:** cronbach’s alpha, eat-26, eating attitude test-26, internal consistency, psychometrics, reliability, validity

## Abstract

The Eating Attitudes Test 26 (EAT-26) scale is a well-established tool for assessing the risk of eating disorders. A reliability generalization meta-analysis was conducted to estimate the average reliability of the EAT-26 scale scores and how reliability estimates vary according to the composition and variability of samples, to identify study characteristics that can explain its variability, and to estimate the reliability induction rate. A literature search produced 14 articles involving 15 studies that met the inclusion criteria. For the total scores of the EAT-26 scale, pooled Cronbach’s alpha was 0.85, with 95% confidence intervals of 0.81 and 0.88, a standard error (SE) of 0.02, and a standard score of (Z = 43.99). Moderator analysis showed that the language, age, or sex of the participants did not affect the overall results. By assessing the reliability of research findings, researchers can examine the consistency of results across studies, which can help identify sources of variability in the results.

## Introduction and background

Eating disorders (EDs) have been defined as the persistent disturbance of eating or eating-related behavior that results in the altered consumption or absorption of food, which significantly impairs health or psychosocial functioning by the Diagnostic and Statistical Manual of Mental Disorders, Fifth Edition (DSM- 5) [1]. They can be severe conditions that impair social, psychological, and physical functions [2]. Types of EDs include anorexia nervosa (AN), bulimia nervosa (BN), binge eating disorder (BED), avoidant restricted food intake disorder, pica, and rumination disorder.

Several countries have seen an increase in the prevalence of EDs in recent decades [3]. In Western countries, the prevalence of AN ranges from 0.5% to 1%, whereas BN ranges from 1% to 2%, and BED ranges from 1% to 3.5% [4]. According to the literature, the point prevalence of EDs has increased over the past few years, rising from 3.5% in 2000-2006 to 7.8% in 2013-2018 [5]. AN has the second-highest fatality rate of any mental health disorder [6]. These findings highlight the importance of EDs as a public health concern and strongly support additional research into ED prevention, detection, and treatment.

Several risk factors can give rise to EDs, which commonly develop during adolescence or early adulthood, though they can also develop earlier or later [7]. EDs are caused by a complex combination of genetic, hereditary, physiological, behavioral, psychological, and social variables [8].

EDs have multiple symptoms. Individuals with AN avoid eating or excessively restrict their food intake and may constantly weigh themselves [9]. Binge-purge AN patients also significantly restrict the amount and kind of food they consume and suffer periods of binge eating and purging [9]. BN is characterized by repeated and frequent uncontrolled episodes of eating excessive amounts of food followed by compensatory behaviors such as vomiting or taking laxatives [9]. The Eating Attitudes Test 26 (EAT-26) scale is an essential tool because it captures a wide array of eating disorders, ensuring a comprehensive assessment of symptomatology across various conditions.

The EAT 26 scale, released in the late 1970s, is a self-reported eating habits assessment tool that includes 26 items for evaluating ED symptoms and concerns; however, because it is self-reported, it is not used as the only basis for diagnosis [10,11]. The EAT-26 scale is broken down into three sections: (a) self-reported height and weight to calculate BMI; (b) 26 items rated on a six-point Likert scale to determine how frequently a person engages in specific behaviors (e.g., “always,” “usually,” “often,” “sometimes,” and “never”); and (c) five behavioral items rated on a similar six-point Likert scale to determine how frequently a person has engaged in disordered eating behaviors in the previous six months [5]. Responses to questions 1- 25 are scored on a four-point scale, with “always” earning 3 points; “usually” earning 2 points; “often” earning 1 point; and “sometimes”, “rarely,” and” “never” receiving 0 points. Reverse scoring is applied to item 26 before adding items 1-26 to determine the final score [5].

The more comprehensive EAT-40 scale has been frequently used to investigate eating-related issues because of the multiple aspects it evaluates and its strong psychometric qualities [4]. In the first version of the EAT-40 scale, seven criteria were added: food obsession, drive for thinness and body image obsessions, misuse of laxatives and vomiting, dieting, slow eating, hidden eating, and perceived pressure to put on weight. A cutoff value of 30 was used to distinguish between participants with EDs and those with regular eating patterns when participants were scored on a six-point Likert scale. The EAT-40 scale may be used to distinguish between AN patients and community-based controls. However, this approach produced a high percentage of false-positive results in potentially high-risk groups: 29% in dance students and 27% in modeling students. Despite these problems, the EAT-40 scale is believed to be a reliable screening tool for identifying people who are generally at risk of developing EDs [4].

After several studies, the questionnaire was reduced to just 26 items. The EAT-26, a shortened version, includes three additional dimensions of bulimia, food preoccupation, and oral control, which are associated with self-control overeating and the perception of pressure from others to gain weight. The answers are graded on a six-point Likert scale, with a cutoff of 20 points. People who score ≥ 20 points are believed to have a generalized pattern of disturbed eating, and both cutoff values (20 for the EAT-26 and 30 for the EAT-40) have been validated by studies using clinical and nonclinical samples [4].

Various ethnic groups (e.g., Arabs, Chinese, Spanish, and others) have been tested with the EAT-26, which is available in the following languages: Arabic, Japanese, Italian, and Hebrew [4]. There are other available scales for assessing the risk of EDs, including the Eating Disorder Inventory (EDI), the Eating Disorder Examination Questionnaire (EDE-Q), the Eating Attitudes Test (EAT), the SCOFF (Sick, Control, One, Fat, Food) questionnaire, the Body Shape Questionnaire (BSQ), the Eating Disorder Assessment Scale (EDAS), the Binge Eating Scale (BES), the Eating Disorder Examination (EDE), the Yale Food Addiction Scale (YFAS), and the Bulimic Investigatory Test of Edinburgh (BITE) [4].

Screening for EDs is important for the early detection and treatment of EDs and can improve patient outcomes, increase the chance of recovery, prevent serious health problems, prevent the development of chronic EDs, reduce the need for intensive treatment, and address mental health issues by preventing patients from worsening [4]. The diagnostic criteria for EDs are specified in the DSM-5, published by the American Psychiatric Association [12].

Meta-analyses are a type of statistical analysis that can help clarify the causes and effects of EDs and can help researchers and clinicians alike make more informed decisions. The reliability and validity of psychological tests, including psychometric tools, are important because they allow for better comparisons across individuals and different populations. One type of study used to determine reliability and validity is a generalizability meta-analysis. Moreover, a meta-analysis is used to gain more scientific power, which increases the likelihood of classifying a genuine impact as statistically significant. The likelihood of identifying a minor impact increases when many studies are merged because many individual studies are too small to be merged in this manner. This approach also increases accuracy. When additional data are used, the estimation of an intervention’s impact can be enhanced [13]. Meta-analyses are used to respond to inquiries that individual studies did not raise [14]. Finally, meta-analyses resolve disagreements resulting from studies that appear to be at odds with one another or to develop new theories. The degree of conflict can be systematically examined by statistical analysis, and the causes of various results can be investigated using a sample of research derived from different studies that share these qualities. Additionally, the causes of variations in effect estimates can be investigated using a sample of different studies that share these qualities [15].

In this review, we present a reliability generalization meta-analysis of one of the most commonly used psychometric tools for assessing EDs in adolescents. We hope this work will assist in guiding future researchers in choosing the most appropriate tool.

## Review

Methodology

Protocol Registration

Review methods and reporting were conducted according to the recommendations of the Reliability Generalization Meta-Analysis (REGEMA) of the EAT-26 scale, which is a type of psychometric meta-analysis that incorporates reliability coefficients from various test applications to different samples (https://osf.io/5ezw2)[16]. In order to ascertain whether reliability can be generalized to various contexts, topics, and demographics, or to identify other aspects of the studies that are statistically linked to the reliability of the test scores, the average reliability of the test scores is to be estimated.

Study Selection Criteria

**Figure 1:**
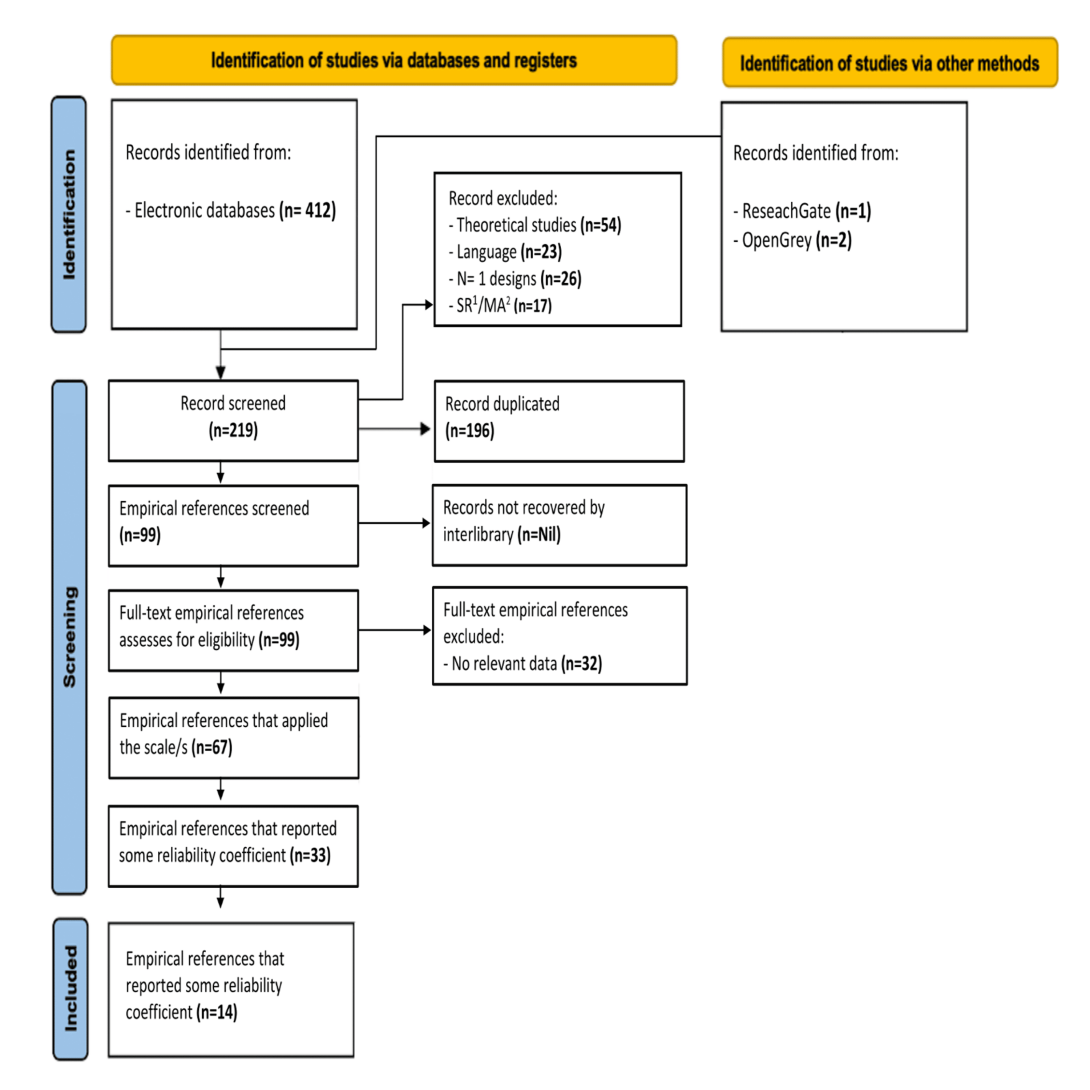
REGEMA flowchart REGEMA: Reliability Generalization Meta-Analysis; SR: Systemic Reviews; MA: Meta-analyses.

Inclusion criteria for studies included in this review were that their authors (a) applied the EAT-26 scale and excluded other EAT scale variations (e.g., EAT-18, EAT-40, EAT-5), (b) imposed no geographical or cultural constraints, (c) selected study dates ranging from 1979 to 2022, (d) wrote in English, French, or Spanish, (e) published their papers, (f) presented reliability estimates based on the study sample, (g) measured internal consistency using Cronbach’s alpha, and (h) focused on a target population (e.g., community, clinical, subclinical, university students) (Figure 1).

Figure 1 illustrates the systematic process of identifying and screening studies for inclusion in research through database and register searches, as well as other methods. Initially, 412 records were identified from electronic databases, and three additional records were sourced from ResearchGate and OpenGrey. During the identification phase, 196 duplicate records were removed, and 120 records were excluded based on criteria such as theoretical studies, language issues, N=1 designs, and systematic reviews/meta-analyses. After the screening, 99 empirical references were further assessed for eligibility, with 32 excluded due to the lack of relevant data. Ultimately, 67 references applied the relevant scales, and 33 of these reported reliability coefficients, 14 references including 15 studies which were included in the final analysis.

Searching for Studies

A systematic search of the following electronic databases yielded relevant articles: PubMed/MEDLINE (Medical Literature Analysis and Retrieval System Online), Embase, PsycINFO, and Scopus. The search strategy included the keywords “reliability”, “validity”, “psychometric”, “internal consistency”, "Cronbach", “Alpha”, “EAT-26”, and “Eating Attitudes Test-26”, which were used to find the full texts of the participants. No search parameters were set.

Data Extraction

The extracted data from the studies included the names of the author or authors, the serial number, publication year, language of the EAT-26 scale applied, sample size, sample description (age and sex), Cronbach’s alpha score, targeted population, and citation.

Reliability Estimates as Outcomes

Internal consistency was assessed using Cronbach’s alpha, which is the type of reliability coefficient employed in this meta-analysis.

Reliability of Data Extraction

Reliability is defined as the consistency or accuracy of measurements [17]. It is a form of validation and is important because, without reliability, we cannot know the true value of an outcome [17]. The most reliable measures are consistently accurate across time and place [17]. Reliability is measured by calculating Cronbach’s alpha, a statistical measure of internal consistency, or the degree to which items within a test measure the same concept [18,19]. The reliability coefficient ranges from 0.00 (inconclusive reliability) to 1.00 (excellent reliability).

Reliability is an important component of the construct validity of a test [20]. Before using the results of a test to make a decision or serve as a measure of a variable, it is critical to examine its reliability, especially when conducting research with populations vulnerable to mental health disorders or those that have cultural or language differences that can influence the measurement of a test [20].

Reliability is a measurement of the consistency and stability of a scale. It measures how well a scale measures the same concept and can be applied to different populations. Reliability is a critical component of scales [17]. A reliability meta-analysis, or the process of combining the results of different studies to determine the overall reliability of a test, can help to determine how well a test can be applied to new or previously tested populations. A reliability meta-analysis can help determine the generalizability of available psychometric tools and improve the reliability of a tool’s results [21]. The random effect model was the statistical model used in the meta-analysis to estimate the average reliability coefficient.

Heterogeneity Assessment

We assessed the heterogeneity among studies by using tau2. If the genuine effect sizes were assumed to be normally distributed, tau was used as an estimate of the standard deviation of the distribution. To calculate the prediction interval, tau was employed. Publication bias was assessed visually using funnel plots; it was assumed that small studies are more susceptible to publication bias than large studies and that this difference is detectable from the graph. In addition, a formal test for asymmetry of the funnel plots was performed using the Fail-Safe N Test, Kendall’s Tau Test, and Egger’s Test.

Software

Statistical analyses were performed using R software, version 4.3.0, released on April 21, 2023 (R Foundation for Statistical Computing, Vienna, Austria). A p-value below 0.05 was deemed statistically significant. The packages employed included "meta" and "metafor".

Results

The targeted population of all studies included university students, AN patients, female soldiers, school students, high school students, ED patients, and healthy community members [22-35]. All the inclusion criteria focused on (a) the use of Cronbach’s alpha for reliability, (b) a population age group of “teenagers” 12-16 years or older, (c) female and male genders, and (d) fluency in the language in which the EAT-26 is administered with a sample size > 90 [22-35].

Fifteen studies [22-35] used the EAT-26 scale translated into 11 different languages (English, Spanish, Arabic, Persian, Hebrew, Chinese, Italian, French, Portuguese, Korean, and Zulu) and provided reliability coefficients based on the data summarizing the internal consistency of EAT-26 scale (see Table 1).

**Table 1:** Summary of internal consistency of EAT-26 scale EAT-26: Eating attitudes test 26; ED: Eating disorder; UAE: United Arab Emirates; PMID: PubMed reference number; CIDI: Composite international diagnostic interview.

SN	PMID/Reference	First Author (Year of publication)	Language of EAT-26 used	Sample size	Cronbach’s Alpha	Target population	Female percentage	Age of subjects (years), mean±SD	Comment
1	20977051 [22]	Rivas T (2010)	Spanish	Study 1: 778	Study 1 α: 0.904	Study 1: Community	100% in both	Study 1: 15.62±2.03	Spanish females in mid-adolescence engage in disordered eating behaviors with high frequency.
2	20977051 [22]	Rivas T (2010)	Spanish	Study 2: 156	Study 2 α =0 .938	Study 2 : clinical and community	100% in both	Study 2: 18.7±4.05	Spanish females in mid-adolescence engage in disordered eating behaviors with high frequency.
3	28994215 [23]	Kang Q (2017)	Chinese	802	0.822-0.922	Clinical (eating disorders patients and healthy individuals)	100%	Clinical group:13-29 years, non-clinical: 18.35±2.67 years	Aimed to investigate the reliability and validity of the Chinese version of EAT-26 among female adolescents & young adults.
4	27745730 [24]	Constaín GA (2017)	Spanish	114	0.89	Community and clinical	0% (Male 100%)	Cases group=21 years; Control group=22 years	Spanish study The aim is to evaluate the validity and diagnostic utility of EAT-26 scale for the evaluation of the risk of eating disorders in the male population.
5	16258635 [25]	Nunes MA (2005)	Portuguese	163	0.75	Clinical (eating disorders patients) and healthy	100%	24.2±4.0	CIDI shows more sensitivity, specificity and positive predictive value than EAT-25 among a sample of Brazilian females.
6	16633489 [26]	Szabo CP (2004)	Zulu	361	0.61	Community	100%	17.87±2.77	Abnormal eating attitudes exist in the rural setting is less than in the urban setting.
7	24703389 [27]	Constaín GA (2014)	Spanish	136	0.92	Community and clinical	100%	Cases group =19 years; Control group= 21 years	To evaluate the validity and diagnostic utility of the EAT-26 scale for the evaluation of the risk of ED in the female population.
8	24563207 [28]	Ahmadi S (2014)	Persian	561	0.82	Community (university students)	100%	21.5 ± 3.44	The analyses of the EAT-26’s divergent and convergent validity showed that pathological eating attitudes and behaviors (except restrained eating) were weakly associated with elevated scores on the BDI-II and BAI
9	10728170 [29]	Dotti A (1998)	Italian	1277	0.86	High school students	81.7%	13-19	The EAT-26 score of girls is significantly higher than that of boys (the difference between the two sexes is approximately half of the mean EAT score of girls.
10	6961471 [30]	Garner DM (1982)	English	300	0.87	University students (comparison group), anorexia nervosa patients	100%	Comparison group=20.3 years, Anorexia nervosa patient=21.5 years	Many female university students have anorexia nervosa.
11	32895080 [31]	Haddad C (2021)	Arabic	811	0.90	Community	65.50%	27.59±11.76	To validate an Arabic version of EAT-26 & identify factors that might be associated with eating disorders.
12	1545342 [32]	Koslowsky M (1992)	Hebrew	809	0.83	Community (female soldiers)	100%	18-19	EAT-26 is reliable for the nonclinical population
13	8193998 [33]	Leichner P (1994)	French	707	0.86	Community (High school student)		14.5±1.5	French study The French version of EAT-26 presents similar characteristics to the English version with a clinical population and a non-clinical population.
14	24688898 [34]	Pourghassem B (2011)	Persian	1850	0.80	Community (high school students)	100%	16.46±1.09	The results are lower than neighboring Arabian countries (Saudi Arabia, Oman, and UAE), but it is higher than Turkey and China.
15	[35]	Rhee MK (1998)	Korean	3496	0.81	Community (Nationwide general population)	40.7% Male, 59.3% Female	18 years and older	Korean EAT-26 is a reliable and valid scale for evaluating disordered eating behaviors and eating problems.

One out of the 15 studies in our meta-analysis included two or more different sample sizes and reliability coefficients [22]. One study included two Cronbach’s alpha values [23]. The total sample size was 12,321 participants, with a mean sample size of 821.4. The mean age of the 15 studies was about 20 years. Ten of the 15 studies [22,23,25-28,30-34] used females only, whereas the remaining three studies [29,31,35] used both men and women, and only one study used men only [24]. The pooled Cronbach’s α was 0.85 (0.81-0.88) with a standard error of 0.02 and a standard score of (Z = 43.99).

**Figure 2:**
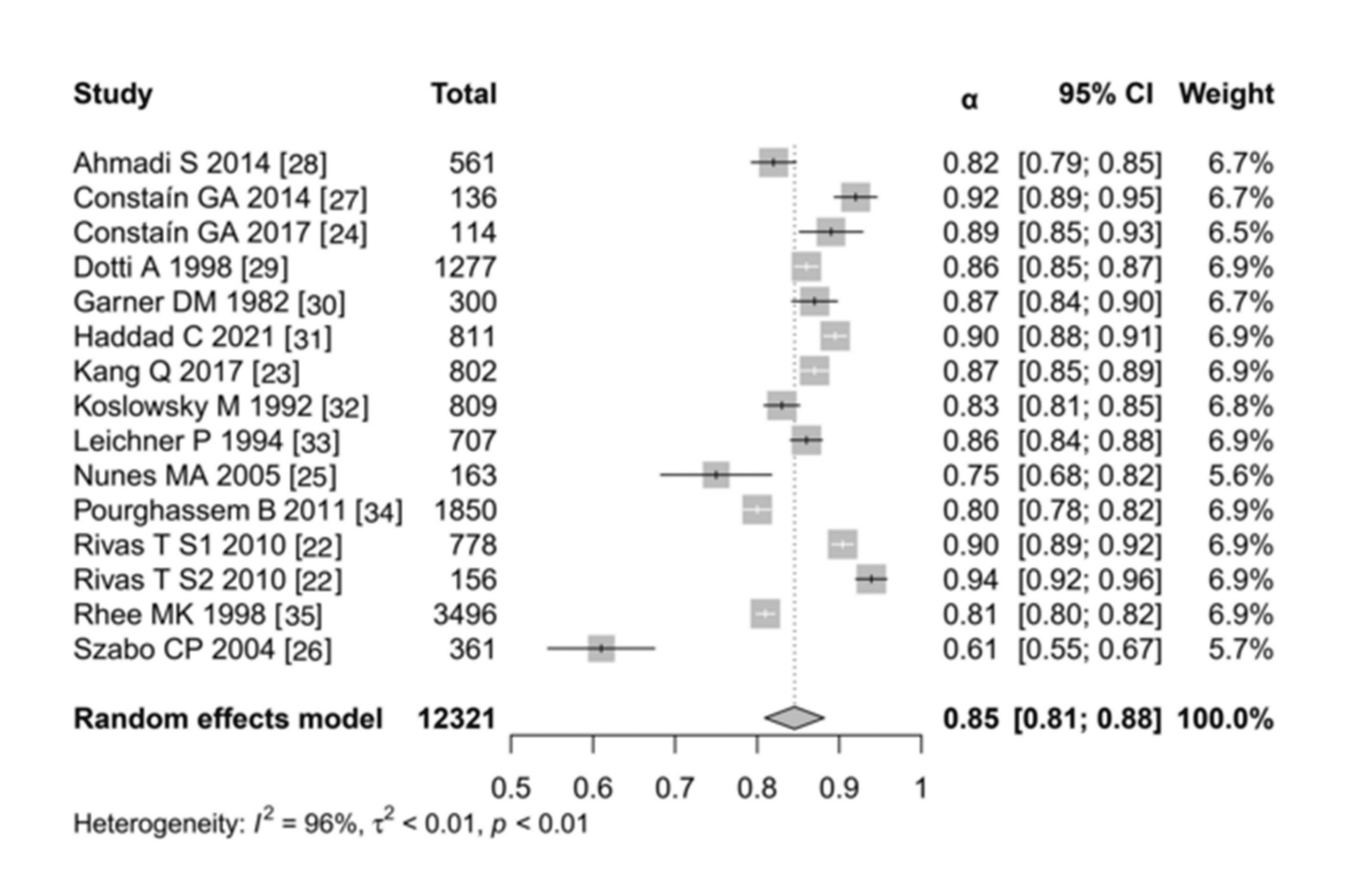
Forest plot of the alpha coefficients reported in the studies on the EAT-26 scale EAT-26: Eating attitudes test 26

According to the model-fit statistics and information criteria, the maximum likelihood of the log-likelihood was 17.55, the deviance was 71.28, and the Akaike information criterion (AIC), which is an estimator of prediction error and thereby, the relative quality of statistical models for a given set of data, was -31.11. The Bayesian information criterion (BIC), which is a criterion for model selection among a finite set of models and is based, in part, on the likelihood function and closely related to the AIC, was -29.69. The AIC and the corrected AIC (AICc) was -30.11. However, in the restricted maximum likelihood, the log-likelihood was 15.87, the deviation was -31.75, the AIC was -27.75, the BIC was -26.47, and the AICc was -26.66. Based on these values, the model perfectly fits the data. The forest plot of the alpha coefficients reported in the studies on the EAT-26 scale is shown in Figure 2.

High heterogeneity was found among the reliability coefficients for the total test score, with T = 0.07, T2 = 0.0053, and I2 = 98%, representing considerable heterogeneity. H2 was equal to 58.80, degrees of freedom (df) = 14, Q test = 372.39, and P < 0.001.

Of the 15 studies included, four [22,27,31], in which the Rivas et al. (2010) paper [22] included two studies, had excellent Cronbach’s α values of 0.9-0.97 that varied among two languages. Eight studies [24,28-30,32-35] had good Cronbach’s α values of 0.8-0.89, one study [25] had acceptable Cronbach’s α values > 0.7, and one study had both excellent and good Cronbach’s α values of (0.822-0.922), except for the one study [26] in a rural, Zulu-speaking, adolescent population in South Africa, which had a questionable Cronbach’s α equal to 0.61. In this study, they hypothesized that the prevalence of abnormal eating attitudes and the potential risk for the development of EDs in a rural setting would be lower than those in an urban setting. In addition, the author built his study based on comparisons with studies performed in North India and Pakistan [26].

Table 2 depicts the replication bias assessment, which employs three different tests: the Fail-Safe N test using the Rosenberg approach, Kendall’s tau test, and Egger’s regression. The first yielded a value of almost 585693 (p < 0.001), but the second and third were -0.16 (p = 0.435) and ‐3.27 (p < 0.001), respectively.

**Table 2:** Replication bias assessment

Test Name	Fail-safe N calculation Using Rosenthal Approach	P	Level of significance
Fail-Safe N	585693	< 0.001	Highly significant
Kendalls Tau	-0.16	0.435	Not significant
Egger’s Regression	-3.27	0.001	Highly significant
Trim and Fill Number of Studies	0.00		

**Figure 3:**
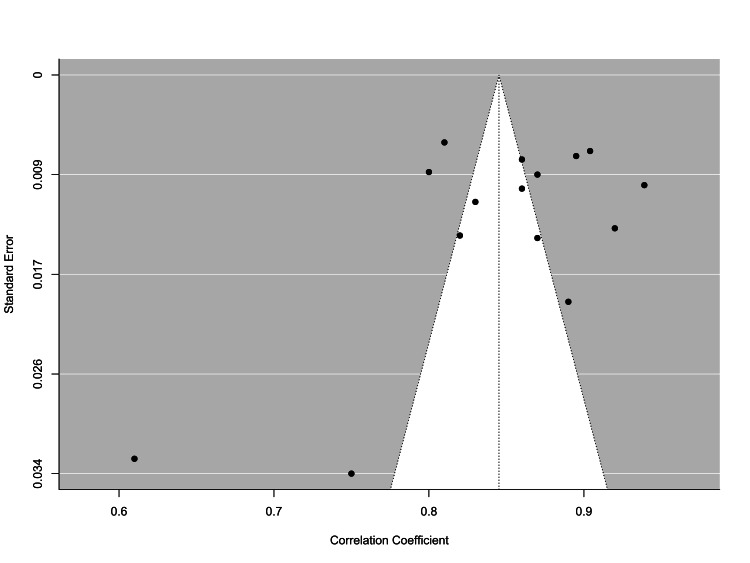
Contoured funnel plot of the alpha coefficients reported in the studies on the EAT-26 scale EAT-26: Eating attitudes test 26

The illustrated contoured funnel plot of the alpha coefficients reported in the studies on the EAT-26 scale is shown in Figure 3, in which the x-axis represents Cronbach’s α, based on the replication bias assessment, and the y-axis represents the standard error, there was no publication bias in the final results.

Discussion

The main objective of this study is to guide future researchers in choosing the most appropriate tool for screening ED in various groups. The EAT-26 scale is a 26-item self-report questionnaire used to assess attitudes and behaviors related to disordered eating. It was designed to assess the presence and severity of disordered eating in clinical and nonclinical populations [10,11]. The test consists of questions about attitudes and behaviors related to dieting, binge eating, and food preoccupation. It is a reliable and valid tool for assessing disordered eating in both clinical and nonclinical populations [4].

We examined all the translations and all the variations in the scale and found the reliability ranged from acceptable to very good. The highest number of studies, four, was published in Spanish [22,24,27] in which the study by Rivas et al. (2010) [22] included two studies, followed by two in Persian [28,34], with one study in each of the remaining languages Italian [29], English [30], Arabic [31], Chinese [23], Hebrew [32], French [33], Portuguese [25], Zulu [26] and Korean [35]. The clinical and community population (students) was larger than the general population (healthy community).

Four out of the 15 studies had Cronbach’s α values, 0.9-0.97, which varied across two languages. Out of the 15 studies, eight had good Cronbach’s α values, 0.8-0.89. One study had acceptable Cronbach’s α values >0.7, and one study had a Cronbach’s alpha of (0.822-0.922), with the exception of the study performed in the Zulu language in 2004 with a questionable Cronbach’s α value of 0.61. As illustrated in the summary of internal consistency of EAT-26 scale in Table 1, there were nine studies with Cronbach’s α of 0.8-0.9 [24,28-35].

The nine studies having Cronbach’s α of 0.8-0.9 mentioned the age group of the target population of 13 years and older; the remaining six studies had broader age groups. Two studies out of the latter six, one published in 2014, were conducted in Spanish with a Cronbach’s alpha of 0.92 with female participants 19-21 years old [27].

The second study [23] was published in Chinese in 2017, with an age group of 13-29 years old. The remaining studies with a Cronbach’s alpha of 0.6-0.7 had an age group of 16-17 years old. The study with the oldest age group in our review used 29-year-olds and was [23] conducted in Chinese in 2017 with a Cronbach’s alpha of (0.822-0.922). The smallest Cronbach’s alpha result was 0.61 for a study done in the Zulu language in 2004 [26] with 17-year-old participants. All these variations in the age groups showed that the age group of the studies’ participants had no effect on the results of Cronbach’s α.

The EAT-26 review concentrated on the reliability (internal consistency) of a single questionnaire across multiple investigations, as demonstrated by a published comparison [36], of the EDE and EDE-Q Meta- Analysis with EAT-26 scale. The EDE vs. EDE-Q study, on the other hand, examined the convergence and differences between an assessment that was questionnaire-based and one that was interview-based. With a Cronbach's alpha of 0.85, the EAT-26 scale demonstrated strong reliability that was constant across demographics. Although the EDE-Q and the EDE interview examine similar constructs, the study found differences in the scores between the two, indicating that although they are reliable, they should not be used interchangeably. Age, sex, and language did not significantly affect reliability, according to the EAT-26 study. The EDE vs. EDE-Q study emphasized the variations in scoring between the two formats (interview vs. questionnaire) rather than concentrating on demographic variables. In conclusion, the EDE and EDE-Q should be used cautiously because they produce somewhat variable results depending on the format utilized, but the EAT-26 scale is a trustworthy and consistent instrument across investigations [36].

Strengths and limitations

This study has a few limitations. First, the studies may have been conducted with different populations and may have collected different outcomes at different time points or used different measures, which can make combining the studies in a meta-analysis difficult, if not impossible. Second, some studies were definitely not found during the meta-analysis search, which may have influenced the results.

This scale is reliable for clinical application. Early screening for EDs can help identify individuals who are at risk for developing an ED and can also facilitate early interventions to help prevent the development of more serious disorders [4]. Likewise, early screening can help identify people who are already showing signs of an ED so they may receive treatment to reduce the severity of the ED [4]. Moreover, early screening can help to reduce the stigma associated with EDs by normalizing their discussion and providing support to those who are struggling [4]. This scale can be compared with other mental state tests (psychiatric examinations) to determine whether the patient has an ED. Finally, it would be helpful to make these findings more accessible at health-care centers for easy diagnosis and screening.

Recommendations for future research

The structure suggested in the current study should be refined and applied to larger samples for confirmatory analysis. Its use in further research involving larger sample sizes, other age groups, or individuals with a clinical diagnosis of ED would provide additional information. To assess the consistency of these encouraging indicators of positive views about one’s body over time, nonclinical and clinical samples must be tested and retested. Future research should consider the development of male-specific risk constructs and models, and future research should test whether the other EAT-scale models are invariant by sex, age, and socioeconomic status.

In this review, we adopted an alpha threshold of 0.7 as the benchmark for reliability, which is in line with established standards in both research and clinical settings [18]. An alpha of 0.7 or higher indicates acceptable internal consistency, ensuring that the scales used are reliable and capable of producing consistent results across different contexts [18]. This level of reliability supports the validity of our findings and underscores the robustness of the instruments employed in our analysis, making them suitable for application in various research and clinical environments.

## Conclusions

Assessing the reliability of research findings enables researchers to evaluate the consistency of results across studies, helping to identify sources of variability. By examining the reliability of their findings, researchers can gain a better understanding of their validity and make more informed decisions regarding the implications of their research. The EAT-26 review shows a pooled Cronbach’s alpha of 0.85, indicating high reliability, supported by confidence intervals ranging from 0.81 to 0.88. Therefore, we conclude that the EAT-26 scale is a trustworthy and excellent tool for use in both clinical and research settings.

## References

[1] Dell'Osso L, Abelli M, Carpita B, Pini S, Castellini G, Carmassi C, Ricca V (2016). Historical evolution of the concept of anorexia nervosa and relationships with orthorexia nervosa, autism, and obsessive-compulsive spectrum. Neuropsychiatr Dis Treat.

[2] Engel SG, Adair CE, Las Hayas C, Abraham S (2009). Health-related quality of life and eating disorders: a review and update. Int J Eat Disord.

[3] Doris E, Shekriladze Ia, Javakhishvili N, Jones R, Treasure J, Tchanturia K (2015). Is cultural change associated with eating disorders? A systematic review of the literature. Eat Weight Disord.

[4] Spivak-Lavi Z, Peleg O, Tzischinsky O, Stein D, Latzer Y (2021). Differences in the factor structure of the eating attitude test-26 (EAT-26) in Different Cultures in Israel: Jews, Muslims, and Christians. Nutrients.

[5] Papini NM, Jung M, Cook A, Lopez NV, Ptomey LT, Herrmann SD, Kang M (2022). Psychometric properties of the 26-item eating attitudes test (EAT-26): an application of rasch analysis. J Eat Disord.

[6] Reese J (2022). Assessment and treatment of eating disorders in adolescents. Contemporary Ob/Gyn.

[7] Smink FR, van Hoeken D, Oldehinkel AJ, Hoek HW (2014). Prevalence and severity of DSM-5 eating disorders in a community cohort of adolescents. Int J Eat Disord.

[8] Treasure J, Schmidt U (2013). The cognitive-interpersonal maintenance model of anorexia nervosa revisited: a summary of the evidence for cognitive, socio-emotional and interpersonal predisposing and perpetuating factors. J Eat Disord.

[9] National Institute of Mental Health . Eating Disorders. https://www.nimh.nih.gov/health/topics/eating-disorders.

[10] Bardone-Cone AM (2007). Self-oriented and socially prescribed perfectionism dimensions and their associations with disordered eating. Behav Res Ther.

[11] Garner DM, Garfinkel PE (1979). The Eating Attitudes Test: an index of the symptoms of anorexia nervosa. Psychol Med.

[12] Regier DA, Kuhl EA, Kupfer DJ (2013). The DSM-5: classification and criteria changes. World Psychiatry.

[13] Cohn LD, Becker BJ (2003). How meta-analysis increases statistical power. Psychol Methods.

[14] Lazonder AW, Harmsen R (2016). Meta-analysis of inquiry-based learning: effects of guidance. Review of educational research.

[15] Cooper H, Hedges LV, Valentine JC (2019). The Handbook of Research Synthesis and Meta-Analysis. https://www.jstor.org/stable/10.7758/9781610448864.

[17] Bannigan K, Watson R (2009). Reliability and validity in a nutshell. J Clin Nurs.

[18] Tavakol M, Dennick R (2011). Making sense of Cronbach's alpha. Int J Med Educ.

[19] Schmitt N (1996). Uses and abuses of coefficient alpha. Psychological Assessment.

[20] John OP, Soto CJ (2007). The importance of being valid: reliability and the process of construct validation. Handbook of Research Methods in Personality Psychology.

[21] Akhter S, Pauyo T, Khan M What is the difference between a systematic review and a meta-analysis?. Basic Methods Handbook For Clinical Orthopedic Research: A Practical Guide And Case Based Research Approach.

[22] Rivas T, Bersabé R, Jiménez M, Berrocal C (2010). The Eating Attitudes Test (EAT-26): reliability and validity in Spanish female samples. Span J Psychol.

[23] Kang Q, Chan RC, Li X (2017). Psychometric properties of the Chinese version of the eating attitudes test in young female patients with eating disorders in mainland China. Eur Eat Disord Rev.

[24] Constaín GA, Rodríguez-Gázquez ML, Ramírez Jiménez GA, Gómez Vásquez GM, Mejía Cardona L, Cardona Vélez J (2017). Diagnostic validity and usefulness of the Eating Attitudes Test-26 for the assessment of eating disorders risk in a Colombian male population (Article in Spanish). Aten Primaria.

[25] Nunes MA, Camey S, Olinto MT, Mari JJ (2005). The validity and 4-year test-retest reliability of the Brazilian version of the Eating Attitudes Test-26. Braz J Med Biol Res.

[26] Szabo CP, Allwood CW (2004). Application of the Eating Attitudes Test (EAT-26) in a rural, Zulu speaking, adolescent population in South Africa. World Psychiatry.

[27] Constaín GA, Ricardo Ramírez C, Rodríguez-Gázquez Mde L, Alvarez Gómez M, Marín Múnera C, Agudelo Acosta C (2014). Diagnostic validity and usefulness of the Eating Attitudes Test-26 for the assessment of eating disorders risk in a Colombian female population (Article in Spanish). Aten Primaria.

[28] Ahmadi S, Moloodi R, Zarbaksh MR, Ghaderi A (2014). Psychometric properties of the Eating Attitude Test-26 for female Iranian students. Eat Weight Disord.

[29] Dotti A, Lazzari R (1998). Validation and reliability of the Italian EAT-26. Eat Weight Disord.

[30] Garner DM, Olmsted MP, Bohr Y, Garfinkel PE (1982). The eating attitudes test: psychometric features and clinical correlates. Psychol Med.

[31] Haddad C, Khoury C, Salameh P (2021). Validation of the Arabic version of the Eating Attitude Test in Lebanon: a population study. Public Health Nutr.

[32] Koslowsky M, Scheinberg Z, Bleich A, Mark M, Apter A, Danon Y, Solomon Z (1992). The factor structure and criterion validity of the short form of the Eating Attitudes Test. J Pers Assess.

[33] Leichner P, Steiger H, Puentes-Neuman G, Perreault M, Gottheil N (1994). Validation of an eating attitude scale in a French-speaking Quebec population (Article in French). Can J Psychiatry.

[34] Pourghassem Gargari B, Kooshavar D, Seyed Sajadi N, Safoura S, Hamed Behzad M, Shahrokhi H (2011). Disordered eating attitudes and their correlates among Iranian high school girls. Health Promot Perspect.

[35] Rhee MK A standardization study of the Korean version of eating attitudes test-26 I: reliability and factor analysis (Article in Korean). Korean Journal of Psychosomatic Medicine.

[36] Berg KC, Peterson CB, Frazier P, Crow SJ (2011). Convergence of scores on the interview and questionnaire versions of the Eating Disorder Examination: a meta-analytic review. Psychol Assess.

